# How to lose your memory without losing your money: shifty epistemology and Dutch strategies

**DOI:** 10.1007/s11229-024-04516-z

**Published:** 2024-04-15

**Authors:** Darren Bradley

**Affiliations:** Philosophy Dept Woodhouse Lane, Leeds University, Leeds, LS2 9JT UK

**Keywords:** Dutch strategies, Memory loss, Learning, Shifty epistemology, Subject-sensitive invariantism

## Abstract

An objection to shifty epistemologies such as subject-sensitive invariantism is that it predicts that agents are susceptible to guaranteed losses. Bob Beddor (Analysis, 81, 193–198, 2021) argues that these guaranteed losses are not a symptom of irrationality, on the grounds that forgetful agents are susceptible to guaranteed losses without being irrational. I agree that forgetful agents are susceptible to guaranteed losses without being irrational– but when we investigate why, the analogy with shifty epistemology breaks down. I argue that agents with shifty epistemologies are susceptible to guaranteed losses in a way which is a symptom of irrationality. Along the way I make a suggestion about what it takes for an agent to be coherent over time. I close by offering a taxonomy of shifty epistemologies.

## Introduction

Some subject-sensitive invariantists hold that one’s evidence shifts with the stakes (Stanley, 2005). Call such views *shifty epistemologies*, and the agents they posit *shifty agents*.^1^ Some object that shifty agents would face guaranteed losses because they are susceptible to Dutch strategies (Greco 2013; Rubin, 2015; Schroeder, 2018). Beddor (2021), following Christensen (1991), has argued that Dutch strategies against shifty agents are analogous to Dutch strategies against forgetful agents, and that neither is a symptom of irrationality. I will argue that forgetful agents are *not* susceptible to Dutch strategies but that shifty agents are. The objection to shifty epistemology remains.

It is natural for shifty epistemologists to try to draw an analogy between loss of knowledge/evidence as the stakes rise and loss of memory. This would provide a familiar precedent. But I will argue that the analogy fails. The way in which knowledge/evidence is lost according to shifty epistemology is not at all similar to everyday memory loss, for only in cases of memory loss do agents take their epistemic position to have *deteriorated*. Agents who take their epistemic position to have deteriorated are not susceptible to Dutch strategies because they will *defer* to their earlier selves. This deference does not appear in shifty epistemologies.

So one aim of this paper is to support the objection to shifty epistemology. To do so, we need to address the question of how forgetful agents avoid Dutch books. And this raises the question of how agents who *learn* avoid Dutch books. There has been surprisingly little discussion of this in the literature. I offer a theory of the relation between forgetting, learning, coherence and rationality.

Section 2 introduces Dutch strategies and the puzzles of learning and forgetting. Section 3 shows how learning and forgetful agents avoid Dutch strategies and Sect. 4 explains how time-slices which fail to defer are susceptible to Dutch strategies. Section 5 explains the analogy Beddor tries to draw between forgetting and shifty epistemology and Sect. 6 argues that the analogy breaks down. Section 7 argues that the way forgetful agents avoid Dutch strategies cannot be mimicked by shifty epistemology. Section 8 develops a taxonomy of shifty epistemologies based on how they deal with cases where the agent foresees the change in stakes. Section 9 concludes.

We’ll take a bit of time discussing how learning and forgetting agents avoid Dutch strategies before we get to Beddor’s argument in Sect. 5. It is worth putting in this time because the issues are important and under-discussed in the literature. Furthermore, it will enable us to move quickly when we get to Beddor’s argument.

## Dutch strategies and the problem of differing credences

A *Dutch book* is a set of bets which are considered fair by the bettor but which guarantee a net loss. A *Dutch strategy* is a set of bets considered fair by the bettor and made at *different times* but which guarantee a net loss.^2^ As it is standardly assumed that an agent who is susceptible to a Dutch strategy is irrational, Dutch strategies have been used to defend various principles about the rationality of particular changes in credences over time.^3^

But the irrationality of susceptibility to Dutch strategies was challenged by Christensen (1991).^4^ He argued that being susceptible to a Dutch strategy was not a symptom of irrationality on the grounds that any two differing credences are susceptible to a Dutch strategy yet such differences are not always symptoms of irrationality.

Let’s briefly rehearse his argument. Start with two different agents. Whenever two agents have different credences, a Dutch book can be made against the pair. The reason is that two people who have different credences will be happy to bet against each other, with each expecting the bet to be favourable to themselves. So both will be willing to pay a bookie a small amount to facilitate the bet. This is a Dutch book against the pair.

The principle is the same if the two differing credences are those of one agent at two different times. The time-slices of the agent will be happy to bet against each other, with each time-slice expecting the bet to be favourable towards themselves. Imagine that a bookie facilitates this bet for a small fee. The agent is susceptible to a Dutch strategy.

Let’s work through an example. Suppose the bettor’s initial credence that tomorrow will be cloudy (p) is 0.5 and their later credence is 0.9. At the earlier time, they would be happy to bet on it not being cloudy (-p) at even odds. That is, they will pay £5 for a ticket that pays back £10 if it turns out to not be cloudy. But after their credence goes to 0.9 they will be keen to pay for a ticket that wins if it is cloudy. So they will be happy to pay £9 for a ticket that pays back £11 if it is cloudy. These two bets constitute a Dutch Strategy: (Table 1).

**Table 1:** Bet payoffs

	Cloudy (P)	Not Cloudy (-P)
Bet 1	-£5	+£5
Bet 2	+£2	-£9
Net	-£3	-£4

So an agent whose credences change over time appears to be susceptible to a Dutch strategy; the only way to avoid being susceptible to a Dutch strategy is to never change credences. It is absurd to claim that one’s credences should never change, so Christensen concluded that being susceptible to a Dutch strategy is not a symptom of irrationality.^5^

But this leaves us with a puzzle. Human beings are forgetful, forgetting results in a shift in credences, so it seems that all humans with imperfect memories are susceptible to a Dutch strategy. If forgetful agents really are susceptible to Dutch strategies, why have we never seen forgetful agents get Dutch strategied? Shouldn’t we expect an industry to exist which profits from people’s forgetfulness? (Compare the way the insurance industry profits from our risk-aversion for large amounts, and the gambling industry profits from risk-lovingness for small amounts.)^6^

The puzzle deepens, for Christensen’s argument applies not just to agents who forget, but agents who learn. When an agent learns, there are two time-slices of the agent with different credences. We can add to the example above so it becomes a case of learning. Suppose that the reason the agent’s credence that it is cloudy goes up from 0.5 to 0.9 is because they learn. They might look at a weather forecast, or wait until the later time and look at the sky. And now it looks like the example shows how an agent who *learns* is susceptible to a Dutch strategy. Do agents who learn really end up with guaranteed losses?!

In the next section I will identify a logically strengthened Dutch strategy which *is* a symptom of irrationality– what I call an explicit Dutch strategy– and show why learning/forgetting agents are not susceptible to it.^7^ I’ll briefly return to Christensen’s argument at the end of Sect. 4.

## How learning and forgetting agents avoid Dutch strategies

Are agents who learn susceptible to Dutch strategies? Surely not. We would expect agents who learn to be *better* off, and have *more* money, than agents who do not learn. I will argue that although there is a technical sense in which learning agents would make bets they consider fair and which guarantee a loss (as we saw in the last section), this is not a loss which is exploitable by a bookie with the same information as the agent.

We first need to understand how a rational agent who learns can find themselves in the position described in the example above. Suppose the proposition at issue is that it is cloudy on Monday (p). On Sunday you have no idea whether it will be cloudy (so your credence is 0.5), but on Monday you see clouds, learn that it is cloudy on Monday and rationally update your credences (to 0.9). On Sunday you might accept the bet that it would not be cloudy (bet 1), but when you see that it is cloudy you expect bet 1 to lose and regret making it. How should you respond? You can minimize losses by betting that it will be cloudy (bet 2). In taking the second bet, you guarantee a loss, but the *expected* loss is less than it would be if you didn’t take the second bet. Given the later credence of 0.9, the expected value of the first bet is (0.9*-5) + (0.1*5) = -4. After making the second bet the expected total loss is only (0.9*-5) + (0.1*5) + (0.9*2) + (0.1*-9) = -3.1. So the guaranteed loss is a result of the agent rationally responding to learning new information and minimizing expected losses.

There is nothing irrational about this sequence of behaviours. But how does a learning agent avoid a sure loss? I think the simplest answer is to note that there is no *explicit* Dutch strategy:

*Explicit Dutch Strategy*.

At all times at which a bet is made, the agent knows the stakes and odds of all other bets that make up the Dutch strategy, and that each bet is part of a Dutch strategy.^8^

Explicit Dutch strategies are most clearly indicators of irrationality. And there is no *explicit* Dutch strategy because you didn’t know the odds of bet 2 when you made bet 1. If you did, you would know that your credence in Cloudy would go up, defer to your later better informed credence, increase your current credence and decline bet 1.^9^*Mutatis mutandis* when credence in Cloudy goes down.

To put the point a bit more generally, for the agent to make a guaranteed loss when their credence in p goes *up* (as in the example above), the first bet must be *against* p and the second bet must be *on* p. To make a guaranteed loss when their credence in p goes *down* (the opposite of the example above), the first bet must be on p and the second bet must be against p. In such cases the agent regrets the first bet and accepts the second bet to minimize expected losses. But knowledge of the odds that they will later consider fair is information about what will happen in the future, which will affect present rational credence. So there is no explicit Dutch strategy against agents who learn.

The same point is sometimes made by positing a bookie and assuming that the bookie has no more information than the bettor. If the bookie has no more information than the bettor then the bookie does not know whether the bettor’s credence will go up or down, so won’t know whether to offer the first bet on p or against p. Thus learning agents are not exploitable by a bookie with the same information as the agent, nor are they irrational.

Forgetful agents avoid explicit Dutch strategies for the same reason, except with the temporal direction reversed. Suppose the earlier credence is 0.9 and, due to forgetting, the later credence is 0.5. At the earlier time they will take bet 2 on it being cloudy (+£2 if cloudy; -£9 if not). Will they take bet 1 (-£5 if cloudy; -£5 if not) at the later time, knowing it completes a Dutch strategy? No. If they know it completes a Dutch strategy then they will be able to work out that the earlier bet must have been on it not being cloudy, and that their earlier, better informed credence that it was cloudy must have been higher than it is now. They should then defer to their earlier higher credence. (They won’t know the exact earlier credence, but knowing that it was higher earlier justifies moving it higher now to some degree). With this higher credence, they will no longer consider bet 1 fair. So there is no explicit Dutch strategy against forgetful agents.

Putting the point in terms of a bookie with no more information than the bettor, neither will remember at the later time whether the earlier credence was high or low, so the bookie won’t know in which direction to offer the later bet (on cloudy or against cloudy?). So forgetful agents are not exploitable by bookies with the same information as the agent.

The key point I will use in my argument against Beddor is: *being forgetful does not make one susceptible to an explicit Dutch strategy.* Before getting to Beddor’s argument in Sect. 5 it is worth expanding on the relation between explicit Dutch strategies, deference, coherence and irrationality.

## Deference, coherence and irrationality

### Deference

Having established that being forgetful does not make one susceptible to an explicit Dutch strategy, we might wonder what does. Specifically, can we identify necessary conditions for being susceptible to a Dutch strategy? In this section I will argue that one necessary condition for being susceptible to an explicit Dutch strategy is that neither time-slice of the agent *defers* to the other. That is, a lack of deference is a necessary (but not sufficient) condition for being susceptible to a Dutch strategy.

One way we get a lack of deference is if the agent considers their shift in credence to be irrational. For example, suppose I am rationally confident that my future high credence that it will be cloudy will be entirely due to a drug that makes people think it is cloudy (and I will be under the influence of this drug whether or not it is cloudy). Assume that the later time-slice thinks the earlier time-slice is irrational. Then my earlier time-slice will rationally bet that it will not be cloudy (bet 1) to reduce the losses expected from later betting that it is cloudy (bet 2). I know the details of both bets at both times, see the loss coming and still make the bets.

Why do I take bet 1? Because it minimizes my expected losses given my later bet and earlier credences. Why do I take bet 2? Because it minimizes my expected losses given my earlier bet and later credences. If I made a bet yesterday and now consider yesterday’s credences to have been irrational, my expected payoff might be negative. I can reduce the expected loss by making a second bet that guarantees a loss over the two bets. Here we have an explicit Dutch strategy. Crucially, there is an explicit Dutch strategy only if neither time-slice defers to the other. If either time-slice defers to the other, the credences at the two times will be identical, and no explicit Dutch book will be possible.

One complication of the drug case is that the later time-slice needs to forget that they have ingested the drug (otherwise they would know that their previous credences were superior). One way to avoid this complication, and indeed a purer example, is to imagine that the agent went through some kind of conversion process that leads them to form different beliefs based on the same evidence. This idea might be most familiar from a religious conversion. We can imagine an agent being sure at the earlier time what will happen, so neither learning nor forgetting, but changing their credences after the conversion.^10^ Each time-slice will fail to defer, so a necessary condition for explicit Dutch strategy susceptibility is in place.

The cases of conversion and drug-ingestion differ from the cases of learning and forgetting regarding deference. Where an agent is sure that their credence will shift in a particular direction due to learning, they should defer to their future self and shift in that direction now. And where an agent is sure that they have shifted in a particular direction due to forgetting, they should defer to their earlier self and shift back. The deference makes them invulnerable to explicit Dutch strategies. Contrapositively, a lack of deference is necessary for being susceptible to an explicit Dutch strategy.

To be clear, having deference does not require *both* time-slices to defer to each other. Having deference requires *agreement* between the time-slices about which is in an epistemically better position. The problem comes only if neither time-slice defers to the other. If your current time-slice thinks it (itself) is best, while the other time-slice thinks it (itself) is best then this disagreement between time-slices produces the susceptibility to an explicit Dutch strategy. We’ll see that this is what happens to shifty agents.

### Coherence

What are the implications for *coherence across time*? Christensen (1991) argued that although agents with different credences at different times are susceptible to an explicit Dutch strategy, there is nothing irrational about that because two time-slices of an agent need not be *coherent across time*.^11^ I want to suggest a definition of coherence across time which we might hope an agent would satisfy.

So, what is it to be coherent across time? One option is to hold that coherence across time requires identical credences over time, but this rules out all learning agents as incoherent across time. Another option is to hold that coherence across time requires updating only by conditionalization, but this rules out forgetful agents as incoherent across time. I suggest that coherence across time requires that *differing times-slices agree on which is in a better epistemic position*, and that the time-slice in the worse position defers to the other. Coherence across time requires deference. Contrapositively, a lack of deference is sufficient for incoherence across time.

**Fig. 1:**
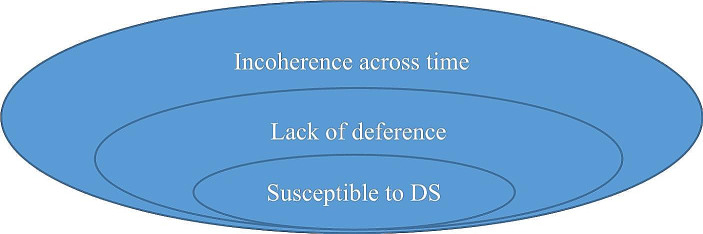
Entailment relations

**Fig. 2:**
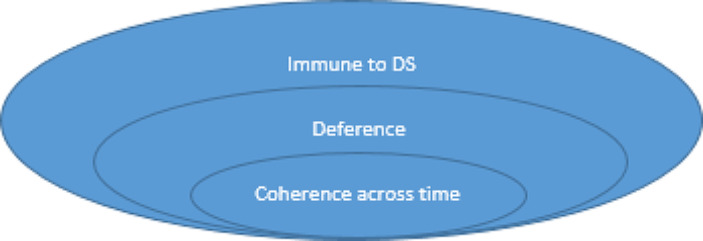
The same entailment relations

Putting this together, I suggest that a lack of deference is a necessary condition on being susceptible to a Dutch strategy (DS) and a sufficient condition for incoherence across time: (See Figs. 1 and 2).

And for those thinking in terms of the negations:

### Rationality

How is this sense of coherence across time related to rationality? One salient hypothesis is: rationality suffices for coherence. I offer three quick arguments that it does not.

First, suppose an agent is certain that their credence in some proposition will shortly be higher due to irrational conversion. It turns out it will be higher due to rational learning. The earlier time-slice does not defer to their later time-slice, so there is incoherence across time, but the agent is perfectly rational at all times.^12^

Second, Permissivists^13^ allow that more than one credence can be rational given a set of total evidence. Permissivists might allow that one can think that one’s own current credence is rational and that a different later credence is rational, even with no evidence learnt between the two times. If so there is rational incoherence across time.

Third, some hold that akrasia (believing that p and that it is irrational to believe p) can be rational. They might allow that one can rationally have one credence while thinking a different credence is rational. Even knowing that they will later have that later credence, they might rationally keep their current credence, so they are rationally incoherent across time.^14^ An improved hypothesis might be: rationality plus certainty that all credence shifts are due to learning or forgetting suffices for coherence. This seems plausible but a detailed discussion would be too great a detour.

It is time to return to Beddor’s argument. To sum up the key points so far, agents are not susceptible to explicit Dutch strategies just because they shift credences e.g. learn or forget. Being susceptible to an explicit Dutch strategy requires that agents shift credences and lack deference.

## Dutch strategies and shifty epistemology

According to shifty epistemology, the truth-values of ascriptions of knowledge, evidence, rational credence or other epistemic notions vary depending on the stakes. Beddor writes:

Start with the idea that knowledge is abundant: we know lots of things about the external world. Combine this with the assumption that knowledge provides a secure basis for action: someone who knows p can rationally act as if p is true. Finally, observe that whether it is rational to act on the basis of some proposition depends on the stakes of the decision. From these premises, subject sensitive invariantists draw the conclusion that knowledge depends on stakes. In ordinary circumstances, knowledge is abundant. But once the stakes go up, it becomes scarce (Stanley, 2005; Fantl and McGrath 2002, 2009; Ross & Schroeder, 2014). (p.2 online)

Beddor extends this argument to apply to evidence, on which we’ll focus:

evidence—like knowledge—provides a secure basis for action… if p is part of your evidence, then it is rational to take p for granted in practical reasoning. Finally, observe that whether it is rational to take p for granted depends on the stakes. (p.2 online)

It follows that when the stakes go up, you have less evidence.

Greco (2013), Rubin (2015) and Schroeder (2018) argue that shifty epistemology must be rejected because it would lead to agents being (explicitly) Dutch bookable. Schroeder gives a nice example:

*Ferris Wheel*.

Sam is riding a Ferris wheel at the carnival, and she is secured in her seat by a seatbelt. While she is at the bottom of the wheel, it is rational for her to act as if the seatbelt will hold her weight if she leans out, because the worst thing that can happen is that she would fall four feet. But when she is at the top of the wheel, it is not rational for her to act as if the seatbelt will hold her weight if she leans out, because if that is false, then she could fall to her death. (2018 p.301)

Shifty epistemology predicts that Sam has evidence that the seatbelt will hold her weight when she is at the bottom of the wheel, but does not have such evidence when she is at the top of the wheel. Sam would bet on the seatbelt holding her weight at the bottom when she has evidence it will, then bet on the seatbelt not holding her weight at the top, when she has no such evidence (at odds which would generate an explicit Dutch strategy). Assuming neither time-slice defers to the other (see Sect. 7), Sam is susceptible to an explicit Dutch strategy.

## Beddor’s response and a reply

Beddor replies that this susceptibility to an (explicit) Dutch strategy is not a symptom of irrationality. He claims that being forgetful makes agents susceptible to an (explicit) Dutch strategy. He goes on to argue that forgetful agents are not irrational^15^ and that the (explicit) Dutch strategy against Sam is no more a symptom of irrationality than an (explicit) Dutch strategy against a forgetful agent.^16^

But we saw above that *being forgetful does not make one susceptible to an explicit Dutch strategy.* Thus Beddor’s argument fails. The explicit Dutch strategy against Sam cannot be dismissed as harmless.

This completes my main argument. It remains to consider a response (Sect. 7) and offer a taxonomy of shifty epistemologies (Sect. 8).

## Can shifty epistemologists avoid the explicit Dutch strategy?

One might think that there is an obvious fix given what I say above– the shifty epistemologist might reply that Sam avoids being susceptible to an explicit Dutch Strategy in the same way that forgetful agents avoid being susceptible to an explicit Dutch Strategy.

Consider Sam at the top of the Ferris wheel, having made a bet at the bottom that the seatbelt will hold her weight. She no longer has such evidence, so is no longer confident that the seatbelt will hold her weight. If Sam would now bet that the seatbelt will *not* hold her weight (with odds matching her current higher credence), then she is susceptible to an explicit Dutch strategy.

To avoid the explicit Dutch strategy in the same way that forgetful agents avoid an explicit Dutch strategy, Sam would have to *defer* to her earlier credences, adopt her earlier high credence that the seatbelt will take her weight, and refuse the second bet. Once her credences return to their earlier high value that the seatbelt will take her weight, she can also freely lean out.

But a central motivation behind shifty epistemology is to explain why one should *not* take such risks! The guiding idea is that one should not act as if p when doing so is risky– that’s why shifty epistemologists conclude that one does not have evidence that p when acting as if p is risky.^17^ Any similarly motivated shifty epistemology must predict that Sam should not lean out. So the shifty epistemologist must maintain that Sam does *not defer* to her earlier credences.

The analogy between losing evidence by forgetting and losing evidence due to high stakes breaks down. Shifty epistemologists don’t think one loses evidence in high stakes situations in the same way that one loses evidence by forgetting– they don’t think that there is a deterioration of the agent’s epistemic state. So the loss of evidence in shifty epistemology cannot be assimilated to the loss of evidence in memory loss.

## Relations between high stakes and low stakes time-slices

These considerations raise the question of how shifty agents should think of the relation between their time-slices at different contexts when they are aware of the different contexts. We can identify a taxonomy of four mutually exclusive (non-exhaustive) positions. If the rational credence does not shift, then there is the question of whether the rational credence fits (i) the low stakes context or (ii) the high stakes context. If the rational credence does shift then there is the question of whether the time-slices consider the credences at other time-slices irrational (iii) or rational (iv). Let’s go through the four positions and the challenges they face.

(i) *Rational credence does not shift and at all times is the rational credence which fits the low stakes context*. This is equivalent to the high stakes time-slice deferring to the low stakes time-slice. This is the response we walked through in the previous section, and found that, although it avoids the explicit Dutch strategy, it undermines the motivation for shifty epistemology.
(ii) *Rational credence does not shift and at all times is the rational credence which fits the high stakes context*. This is equivalent to the low stakes time-slice deferring to the high stakes time-slice. This is plausible if we think of shifts in rational credence as being induced by the agent becoming aware of some high-stakes possibility. If so, there will be no explicit Dutch strategy in cases where the agent is always aware of the high stakes possibility. When offered a bet that the seatbelt will hold at the bottom, Sam will foresee that she will soon be in a high stakes context, so will effectively be in a high stakes context already, will have low credence that the seatbelt will hold and refuse the bet.

A similar view is defended by Schroeder (2018) about full belief (but not credence). On his view a belief is a long-term plan for dealing with various situations. If foreseeable situations include high stakes situations then a belief is only rational if it suitably deals with those situations. So if high stakes situations are expected to arise, beliefs should only be formed if they can deal with those high stakes situations. But it is not natural to extend this account to credence (Schroeder does not extend it), as credences are not usually considered long-terms plans for dealing with situations; credences are usually considered to be responses to the evidence.

A further challenge (for both beliefs and credences) concerns whether the agent must be *certain* that they will end up in a high stakes situation. If so, then, given that we are almost never certain of anything that will happen in the future, agents will never rationally defer to the high stakes credence. So suppose instead that we do not require certainty of the future. How confident must the agent be that they will ever be in a high stakes situation? If any non-zero probability is sufficient, then agents will almost always defer to some hypothetical high-stakes context, because there will be some high stakes hypothesis which has a non-zero probability. Perhaps agents should adopt the credences suitable for high stakes contexts if their credence that they will be in a high stakes context is above some middling credence. This faces the challenge of finding a value for the middling credence without arbitrariness. Alternatively, perhaps credences gradually shift towards what they would be in a high stakes context as high stakes contexts become more likely.

(iii) *Rational credence shifts and one (or more) of the time-slices should think another time-slice is irrational.* This fits the example in Sect. 4 of an agent who undergoes a conversion. We can think of Sam at the bottom having high credence that the seatbelt will hold, but having low credence at the top. Sam at the top thinks that the low credence is always rational, while Sam at the bottom thinks the high credence is always rational. So each time-slice thinks the other is mistaken.

As we saw above, this agent is susceptible to an explicit Dutch strategy. A further problem is that this view supposes that Sam is rational in both contexts, but each of Sam’s time-slices believes the other time-slice is irrational. So each of Sam’s time-slices has a false belief about rationality. It is counter-intuitive that rational agents cannot avoid systematic and predictable false beliefs about rationality. Worse, Sam could work through the reasoning of this section and conclude that she is rational in both contexts. So this position looks unstable.

(iv) *Rational credence shifts and each time-slice should consider the other rational.* On this view, Sam at the bottom has high credence that the seatbelt will hold, foresees that at the top she will have low credence that the seatbelt holds, and at both times considers both credences rational. Both time-slices do not defer.^18^

This view faces the problem that, as the time-slices do not defer, the agent is susceptible to an explicit Dutch strategy.^19^

Nevertheless, this view might be attractive to those inclined towards *permissivism*, which permits different credences given the same set of evidence. Some versions of permissivism allow that an agent can consider an alternative credence to their own to be rational, while not deferring to that alternative credence. This might provide a precedent for allowing that each time-slice considers the other rational, but does not defer to it. (One difference is that permissivists allow different credences given the same evidence, but we have been supposing that the evidence differs at the top and bottom. We might think of this as permissivism about the evidence rather than about credence i.e. it is permissible to have different evidence.)

However, the relevant form of permissivism would be *acknowledged intrapersonal* permissivism. In *acknowledged* permissive cases an agent with one permissible credence function can recognize that another credence function is rational (rather than being unaware).^20^ In *intrapersonal* cases, the same agent (rather than different agents) can have different permissible credences. Most defences of permissivism only defend inter-personal permissivism; defending acknowledged intrapersonal permissivism is particularly difficult.

To fill out the difficulties, we can see how the main ways in which permissivism has been defended don’t apply naturally to the case of the Ferris Wheel. One way to defend permissivism is to argue that the different (time-slices of) agents have different goals.^21^ But Sam’s time-slices presumably have the same goal of enjoying the Ferris Wheel without getting hurt. Goals do not seem to change between the top and bottom. Another way to defend permissivism is to argue that the (time-slices of) agents have different starting points.^22^ But in the case of the Ferris Wheel the time-slices have the same starting point i.e. their position before they got on the Ferris Wheel. So permissivism does not provide an easy way out of the difficulties in this case.

I don’t consider any of these four options to be refuted by my comments. But clearly there are difficult questions that shifty epistemologists need to answer.

## Conclusion

I have argued that agents who learn or forget are not susceptible to an explicit Dutch strategy. An explicit Dutch strategy is avoided as long as both time-slices agree about which time-slice is in an epistemically superior position and defers to that time-slice. This undermines Beddor’s defence of shifty epistemology. The agents predicted by shifty epistemology are not analogous to agents who forget. Agents who forget typically defer to their earlier selves; the agents predicted by shifty epistemology do not. So it really is a problem that shifty epistemology predicts that agents will routinely be susceptible to explicit Dutch strategies.

## Footnotes

1 Beddor cites Stanley (2005: 182), Weatherson (2018), and Beddor (2020) as advocates of shifty views of evidence; Fantl and McGrath (2009) and Schroeder (2018) have shifty views of knowledge and belief but do not extend the shiftiness of beliefs to evidence or credences, and so avoid the Dutch book argument.

2 ‘In the diachronic case we should speak of a Dutch strategy rather than a Dutch book’ (van Fraassen, 1984 p.240).

3 A Dutch strategy has been influentially used to argue for conditionalization (Lewis, 1999) and Reflection (van Fraassen, 1984). See Skyrms (1993) for a helpful discussion of Dutch strategies. See Pettigrew (2021) for a recent study and further references.

4 See also Schick (1986), Bacchus et al. (1990), Talbott (1991) and Maher (1992).

5 ‘This suggests, of course, that vulnerability to the Dutch Strategy does not indicate irrationality after all’. Christensen (1991) P.242.

6 To be clear, I don’t think Christensen is committed to there being a Dutch book against forgetting agents; but Beddor takes Christensen’s discussion to suggest that there is. Briggs (2009) also seems to suggest that forgetful agents are susceptible to Dutch strategies.

7 Skyrms (1987) proves that conditionalizing agents are not susceptible to a Dutch strategy. But that does not *explain how* they avoid the strategy described above.

8 See Talbott (1991 p.144-5).

9 This invokes a plausible version of the Principle of Reflection (see van Fraassen, 1984, Weisberg, 2007, Briggs, 2009).

10 The Bayesian can model this conversion in terms of either a change in priors, P(H), or a change in likelihoods, P(E|H), and it won’t matter here which we choose.

11 ‘…[W]ithout independent reason to think that the agent’s future beliefs should mesh in some particular way with her present ones, we have no reason to think she should avoid the *incoherence* exploited by the imaginary bookie.’ Italics added, Christensen (1991) P.242.

12 One might propose that the Dutch strategy susceptibility of shifty agents is also due to thinking they might be irrational in the future. But we’ll see in Sect. 8 (case iii) that this doesn’t really fit what happens to shifty agents. Furthermore, in the Ferris Wheel case Sam is certain what will happen in the future, whereas here ignorance about the future is essential to the incoherence.

13 See White (2005). See Greco and Hedden (2015) on the connection between deference and permissivism. Further references are in Sect. 8.

14 In my view akrasia is sometimes rational (Bradley, 2019). Thanks to Adam Elga for raising akrasia.

15 ‘Contrary to what is often claimed, it is not necessarily irrational to accept a diachronic Dutch book.’ Beddor 2021 p. 198.

16 ‘…[I]n Memory Manipulation, your behaviour is rational… if we say this about Memory Manipulation, we can say the same thing about Stakes Manipulation.’ Beddor 2021 p. 198.

17 This can be seen in the quotes from Beddor above. Similarly, in their seminal paper, Fantl and McGrath (2002) argue that whether you should get on a train depends on how bad the consequences will be if the train isn’t the one you think it is. They argue that if the stakes are high you should not take the risk of getting on the train (without checking if it is the right one), and they use shifty epistemology to explain why not. A referee complains that this just confuses probability with utility. I agree, but that is a matter for another paper.

18 A different view is that each time-slice considers their own credence irrational and the other time-slice rational. But this involves a particularly implausible form of akrasia, and an endless cycle in which t1 defers to t2, which defers to t1… The same applies if each time slice considers their own credence rational and defers to the other.

19 A referee suggests that an agent with a credence of 0.8 in p might get a bop on the head and shift to a credence of 0.7 (while receiving no new relevant evidence). If the agent, at both times, considers both credences rational then they might be rational and susceptible to an explicit Dutch strategy. My inclination is to say that the Dutch strategy offers a vivid demonstration of the arbitrariness problem for permissivism (White, 2005, 2014; see Meacham, 2021 for a recent discussion and further references).

20 See Ballantyne and Coffman (2012, p. 663).

21 See Pettigrew (2022).

22 The literature often talks of ‘standards’ (e.g. White, 2005, Schoenfield, 2014) but this seems to mean ‘starting point’ which in a Bayesian context means ‘prior’.
